# RBMX Transcriptionally Repressed by EZH2‐Associated H3K27me3 Modification Attenuates Pyroptosis in Renal Ischemia/Reperfusion Injury via Regulating SIRT3/NLRP3 Inflammasome Activation

**DOI:** 10.1002/kjm2.70188

**Published:** 2026-03-02

**Authors:** Yi‐Han Wang, Yan Teng, Fang‐Lan Yao, Shu‐Qi Wei, Yan Li

**Affiliations:** ^1^ Department of Kidney Transplantation The First Affiliated Hospital of Xi'an Jiaotong University Xi'an Shaanxi China; ^2^ Emergency Intensive Care Unit The First Affiliated Hospital of Xi'an Jiaotong University Xi'an Shaanxi China

**Keywords:** acute kidney injury, ischemic reperfusion injury, NLRP3 inflammasome, pyroptosis, RBMX

## Abstract

Ischemic reperfusion injury (IRI) to kidney is a significant clinical factor in acute kidney injury (AKI). This study aimed to investigate the new role of RNA‐binding motif protein X‐linked (RBMX), a modulator of m6A methylation, in renal IRI and to examine the associated regulatory mechanisms. An in vitro renal IRI model was established using HK‐2 cells subjected to hypoxia‐reoxygenation (H/R) treatment. To investigate the role of RBMX, RBMX‐overexpressing cells were transfected with pcDNA/RBMX. The viability of HK‐2 cells was evaluated using the CCK‐8 assay. EdU was utilized to evaluate cell proliferation in HK‐2 cells. Western blot analysis was conducted to determine the expression levels of proteins involved in NLRP3 inflammasome activation. ELISA was used to measure the secretion of inflammatory cytokines linked to pyroptosis. LDH and PI staining were used to investigate pyroptosis. IP, RIP, and MeRIP assays were performed to detect NLRP3 acetylation and the interaction between NLRP3 and SIRT3. An in vivo IRI mouse model was established to further validate the renoprotective effect of RBMX. Our results showed that RBMX expression was significantly downregulated in IRI mice and in vitro H/R‐treated HK‐2 cells. In H/R‐induced HK‐2 cells, RBMX overexpression attenuated NLRP3 inflammasome activation and pyroptosis, shown by reduced expression levels of NLRP3, ASC, cleaved caspase‐1, and GSDMD‐N, along with decreased levels of IL‐18, IL‐1β, TNF‐α, and IL‐6. Additionally, RBMX is associated with the m6A methylation of SIRT3, which is involved in the control of NLRP3 acetylation and activation in H/R‐exposed HK‐2 cells. SIRT3 knockdown reversed the impacts of RBMX on cell proliferation, NLRP3 inflammasome activation, and pyroptosis. Moreover, EZH2 may be involved in an upstream gene that mediates the H3K27me3 modification of RBMX. Finally, in vivo assays provided evidence suggesting that RBMX overexpression improved renal injury in mice. Taken together, our data support a potential role of the m6A regulator RBMX in suppressing NLRP3 inflammasome activation and pyroptosis possibly through the regulation of m6A methylation of SIRT3 in renal IRI. We hypothesized that targeting the EZH2/RBMX/SIRT3 axis might represent a new therapeutic approach to impede the progression of renal IRI.

## 1 Introduction

Ischaemia reperfusion injury (IRI) to the kidney is a primary clinical factor leading to acute kidney injury (AKI) [1]. Renal tubular epithelial cells exhibit high sensitivity to IRI due to the anatomical characteristics of kidney tissues [1]. Therefore, minimizing IRI in the kidneys is crucial for AKI prevention and treatment.

Pyroptosis is a form of pro‐inflammatory programmed cell death implicated in IRI [2]. Distinguished from other programmed cell death types, pyroptosis operates via the inflammasome pathway, enhancing the release of inflammatory cytokines [3, 4]. Subsequently, activation of the NLRP3 inflammasome leads to active caspase‐1 formation, ultimately facilitating pyroptosis [5]. NLRP3 inflammasome‐mediated pyroptosis induces the release of IL‐1β and IL‐18, initiating a cascade of inflammatory responses [5]. Therefore, targeting NLRP3 inflammasome‐mediated pyroptosis may be crucial in mitigating or preventing IRI.

Mounting evidence suggests that N6‐methyladenosine (m6A) RNA methylation is implicated in various physiological and pathological processes, thereby contributing to a range of human diseases such as cancers, cardiovascular diseases, and IRI [6, 7, 8]. The X‐linked RNA‐binding motif protein (RBMX) is a nuclear RNA‐binding protein encoded on the X chromosome [9]. Significantly, RBMX is linked to the regulation of m6A methylation and may be one of the important m6A reading proteins [10, 11]. Recently, RBMX was found to be downregulated in ischemic stroke patients [12]. However, its potential role in IRI and the underlying molecular mechanisms have not been elucidated. In this study, we explore a novel function of RBMX in renal IRI and investigate the upstream and downstream molecules along with the regulatory mechanisms involved.

## 2 Methods and Materials

### 2.1 Datasets Collection

Potential upstream regulators of RBMX were obtained from the KnockTF (http://www.licpathway.net/KnockTF/) and CTD databases (http://ctdbase.org/). The GSE34351 dataset was downloaded from the Gene Expression Omnibus database (GEO, https://www.ncbi.nlm.nih.gov/geo/). Data on m6A regulators were obtained from the RM2Target (http://rm2target.canceromics.org/).

### 2.2 Cells Culture and Treatments

The human proximal tubular epithelial cell line (HK‐2; American Type Culture Collection, ATCC Manassas, VA, USA) was cultured in DMEM/F12 medium (Gibco, Grand Island, NY, USA) containing 10% foetal bovine serum (FBS; Gibco) and 1% penicillin/streptomycin (Gibco). The HK‐2 cells were maintained in an incubator with 5% CO_2_ at 37°C. For hypoxia‐reoxygenation (H/R) treatment, HK‐2 cells cultured in serum‐free medium were exposed to hypoxic conditions (94% N_2_, 5% CO_2_, and 1% O_2_) for 12 h. The cells were then reoxygenated (74% N_2_, 5% CO_2_, and 21% O_2_) for 6 h.

To elevate RBMX expression, pcDNA/RBMX or the empty vector pcDNA3 (negative control) was transiently transfected into HK‐2 cells using Lipofectamine 2000 (Invitrogen, Carlsbad, CA, USA). Small interfering RNAs (siRNAs) targeting SIRT3 (si‐SIRT3), si‐EZH2, and si‐con (negative control) were designed and synthesized by GeneCreate Bioengineering (Wuhan, China). Cells were transfected using Lipofectamine 2000 (Invitrogen, Carlsbad, CA, USA). At 48 h post‐transfection, transfection efficiency was confirmed by detecting protein levels by Western blotting.

### 2.3 RT‐PCR

Total RNA was extracted from HK‐2 cells using the TRIzol reagent (Invitrogen), and the purified RNA was reverse‐transcribed into cDNA using a reverse transcription kit (TransGen, Beijing, China). To quantify RBMX mRNA levels, qPCR was performed using a real‐time quantitative PCR kit (TransGen). The 2^−ΔΔCt^ method was applied to calculate the relative mRNA level of RBMX. RBMX primers forward 5′‐CTTCAGGACCAGTTCGCAGTA‐3′, reverse 5′‐TCACGACCACTTGAGTAGAGAT‐3′; EZH2 primers forward 5′‐GTGGAGAGATTATTTCTCAAGATG‐3′, reverse 5′‐CCGACATACTTCAGGGCATCAGCC‐3′; GADPH primers forward 5′‐GGAGCGAGATCCCTCCAAAAT‐3′, reverse 5′‐GGCTGTTGTCATACTTCTCATGG‐3′.

### 2.4 Western Blot Analysis

Whole cell lysates from HK‐2 cells and kidney tissues were prepared using RIPA lysis buffer (Solarbio, Beijing, China). The protein concentration was quantified using a BCA protein detection kit (Boster Biological Technology, Wuhan, China). Proteins were fractionated using 10% SDS‐PAGE and electrophoretically transferred onto PVDF membranes. The membranes were incubated with 5% (w/v) non‐fat milk for 1 h and then incubated with the appropriate primary antibody overnight at 4°C. The primary antibodies include anti‐RBMX, anti‐NLRP3, anti‐ASC, anti‐Caspase‐1, anti‐GSDMD‐N, anti‐SIRT3, anti‐enhancer of zeste homologue 2 (EZH2), anti‐H3K27me3, and anti‐β‐actin (1:1000; Abcam, Cambridge, MA, USA). Subsequently, the membranes were incubated with goat anti‐rabbit secondary antibody (1:3000; Abcam). Immunoreactive bands were detected using an ECL kit (Thermo Fisher Scientific, Waltham, MA, USA), and the blots were analyzed using ImageJ software.

### 2.5 CCK‐8 Assay

Differently transfected HK‐2 cells were seeded into 96‐well plates at a density of 5 × 10^3^ cells/well. Afterwards, 10 μL CCK‐8 solution (Solarbio, Beijing, China) was added to each well and incubated for another 30 min. The absorbance was measured at 450 nm using a microplate reader (Bio‐Tek, Winooski, VT, USA).

### 2.6 5‐Ethynyl‐20‐Deoxyuridine Assay

The proliferative ability of HK‐2 cells was determined using a 5‐ethynyl‐20‐deoxyuridine (EdU) assay kit (RiboBio, Guangzhou, China). Briefly, cells (1 × 10^5^ cells/well) were seeded into confocal plates and then incubated with 50 μM EdU buffer for 6 h at 37°C. The cells were fixed with 4% formaldehyde for 30 min, washed with PBS containing 3% BSA, and permeabilised with 0.3% Triton X‐100 for 20 min. The EdU solution was added to the culture, followed by nuclear staining with Hoechst33342 for 10 min. EdU‐positive cells were visualized under a fluorescence microscope (Olympus, Tokyo, Japan).

### 2.7 ELISA

Conditioned media of HK‐2 cells or serum samples from mice were collected, and the TNF‐α, IL‐1β, IL‐6, and IL‐18 levels were quantified using the respective ELISA kits (Proteintech, Chicago, IL, USA) following the manufacturer's instructions.

### 2.8 Lactate Dehydrogenase (LDH) Release Assay

Transfected HK‐2 cells were added into 96‐well plates at a density of 5 × 10^3^ cells/well and incubated with an LDH assay kit (Beyotime Biotechnology, Shanghai, China). The absorbance at 450 nm was detected with a microplate reader (Bio‐Tek). LDH release was presented as % of the control group.

### 2.9 Propidium Iodide (PI) Staining Assay

Inflammatory cell death was analyzed by PI and Hoechst 33432 staining for 10 min. The cells were observed under a fluorescence microscope. Pyroptotic cells were presented as PI‐positive cells.

### 2.10 Immunoprecipitation Assay

To detect NLRP3 acetylation and the interaction between NLRP3 and SIRT3, immunoprecipitation (IP) assays were performed as previously described [13]. Briefly, HK‐2 cells were lysed in a lysis buffer and centrifuged at 12000 × *g* at 4°C for 10 min. The supernatants were then immunoprecipitated with an antibody against pan‐Ac‐Lys, NLRP3, SIRT3, or IgG (Abcam) for 12 h at 4°C. The following day, magnetic protein A/G beads (Thermo Scientific) were added, followed by 2 h of rotation at room temperature. The immunoprecipitates were washed six times with IP buffer and boiled in SDS‐PAGE loading buffer for Western blot analysis.

### 2.11 RNA Immunoprecipitation Assay

RNA immunoprecipitation (RIP) assay was conducted using the Magna RIP RNA‐Binding Protein Immunoprecipitation Kit (Millipore, Billerica, MA, USA). According to the manufacturer's protocol, HK‐2 cell lysates were prepared and incubated with anti‐RBMX (Abcam) or anti‐IgG antibodies (internal control). After removing the rRNA, the immunoprecipitated RNA was extracted and quantified by RT‐qPCR.

### 2.12 Methylated RNA Immunoprecipitation Assay

The m6A modification level of SIRT3 was measured by methylated RNA immunoprecipitation (MeRIP) assay using GenSeq m6A MeRIP Kit (Cloudseq, Shanghai, China). As previously described [14], RNA from HK‐2 cells was extracted and purified, followed by fragmentation into approximately 100‐nt fragments. Next, the RNA fragments were immunoprecipitated with anti‐m6A or anti‐IgG antibodies (Abcam) together with protein A/G magnetic beads (Thermo Scientific). Antibody‐bound methylated RNAs were eluted and subjected to RT‐qPCR.

### 2.13 Renal Ischaemia/Reperfusion Injury Model in Mice

A total of 32 wild‐type male C57BL/6J mice (aged 6–8 weeks) were purchased from the Experimental Animal Centre of Xi'an Jiaotong University. All procedures followed the Guidelines for the Care and Use of Laboratory Animals and were approved by the Committee on the Use of Live Animals of the First Affiliated Hospital of Xi'an Jiaotong University (Xi'an, China). To construct an IRI‐induced AKI model [15], mice were anesthetized using isoflurane inhalation, followed by a midline abdominal incision to expose the kidneys. Subsequently, the left renal artery and vein were carefully dissected, and the left renal pedicle vessel was clamped using a microvascular clamp (Roboz Surgical Instrument, Gaithersburg, MD, USA) for 45 min to induce ischaemia. Subsequently, the clamp was removed to allow reperfusion, and the abdominal incision was sutured. In the RBMX overexpression group, the mice were administered a recombinant adeno‐associated virus encoding the RBMX gene designed by GenePharma (Shanghai, China) via renal vein injection [15]. At 24 h post‐reperfusion, blood and urine samples were collected for the detection of inflammatory cytokines, creatinine, blood urea nitrogen (BUN) and urinary N‐acetyl‐beta‐D‐glucosaminidase (NAG) levels. The mice were sacrificed by cervical dislocation, and their kidneys were collected for further experiments, including Western blotting and histological detection.

### 2.14 Histological Detection

At the end of the experiments, kidneys were harvested from the mice and fixed in 4% paraformaldehyde. Then the tissues were embedded in paraffin and cut into 5 μm sections, which were subsequently stained with H&E for histological examination by two experienced researchers. The degree of kidney injury was assessed as previously published [16].

### 2.15 Immunofluorescence Staining

HK‐2 cells were fixed in 4% paraformaldehyde for 20 min and permeabilised with 0.3% Triton X‐100 for 10 min. After blocking with 1% FBS for 2 h at 37°C, the HK‐2 cells were incubated overnight with anti‐EZH2 or anti‐H3K27me3 (Abcam) at 4°C. Subsequently, Alexa Fluor 488 or 647 anti‐rabbit IgG (Abcam) was added to the incubation system for 1 h at 37°C. Nuclei were stained with DAPI (Beyotime Biotechnology). Finally, cells were examined under a fluorescence microscope (Olympus).

### 2.16 Statistical Analysis

Data analyses were performed using GraphPad Prism version 8.0.1 (GraphPad Software, San Diego, CA, USA). Student's *t*‐test and one‐way analysis of variance (ANOVA), followed by the Bonferroni post hoc test, were used to assess significant differences between two groups and multiple groups. Statistical significance was set at *p* < 0.05.

## 3 Results

### 3.1 RBMX Expression and Its Role in HK‐2 Cell Proliferation Under H/R‐Conditions

**FIGURE 1:**
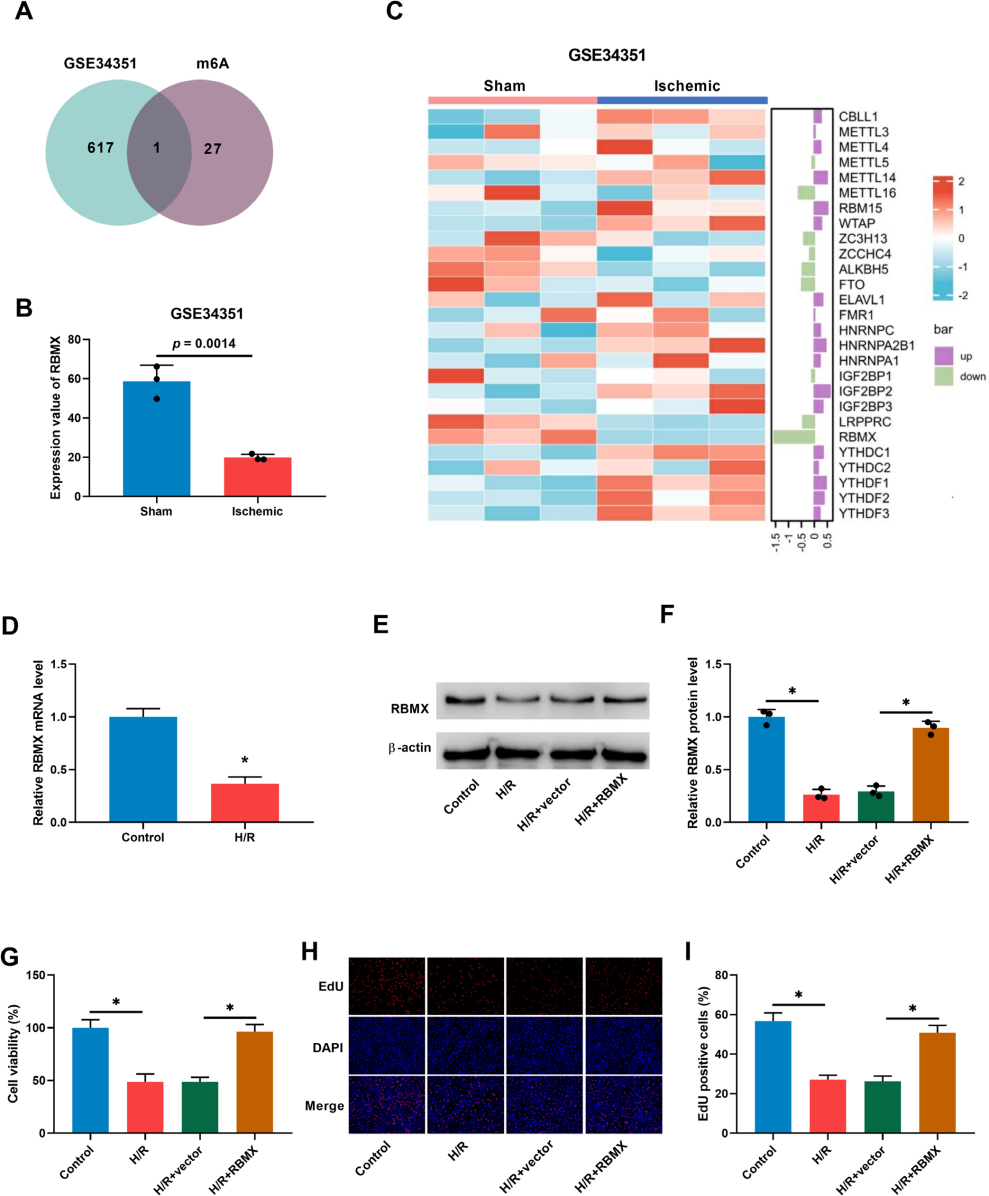
RBMX regulated cell proliferation of HK‐2 cells under H/R condition. (A) Venn analysis of GSE34351 dataset (adj.*p* < 0.05, |logFC| ≥ 1) and m6A regulators. (B) RBMX expression in sham and ischemic mice from GSE34351. (C) Expression of m6A regulators in sham and ischemic mice from GSE34351. (D) RBMX mRNA in H/R‐treated HK‐2 cells and control HK‐2 cells. (E, F) RBMX protein levels in different groups. (G) CCK‐8 assay for cell viability of HK‐2 cells. (H, I) EdU for cell proliferation of HK‐2 cells. * *p* < 0.05.

First, we utilized bioinformatics analysis to identify potential target genes in individuals with ischaemia. Venn analysis revealed that RBMX is an m6A regulator that is differentially expressed in ischaemic mice (Figure 1A). Additionally, RBMX expression was significantly downregulated in the ischaemic group compared to the sham group based on the GSE34351 dataset (Figure 1B). The changes in m6A regulator expression are illustrated in Figure 1C, indicating a significant decrease in RBMX expression in the ischaemic group. In vitro assays were conducted to investigate the role of RBMX in H/R‐treated HK‐2 cells. RT‐PCR and Western blot analyses indicated a substantial downregulation of both RBMX mRNA and protein levels in H/R‐treated HK‐2 cells (Figure 1D–F). To elucidate the precise function of RBMX, RBMX‐overexpressing cell lines were transfected with pcDNA/RBMX. As shown in Figure 1E,F, transfection with pcDNA/RBMX increased RBMX expression in H/R‐treated HK‐2 cells. The CCK‐8 and EdU assays showed that RBMX overexpression mitigated the H/R‐induced decline in cell viability and proliferation (Figure 1G–I).

### 3.2 Effects of RBMX Overexpression on H/R‐Induced NLRP3 Inflammasome Activation and Pyroptosis in HK‐2 Cells

**FIGURE 2:**
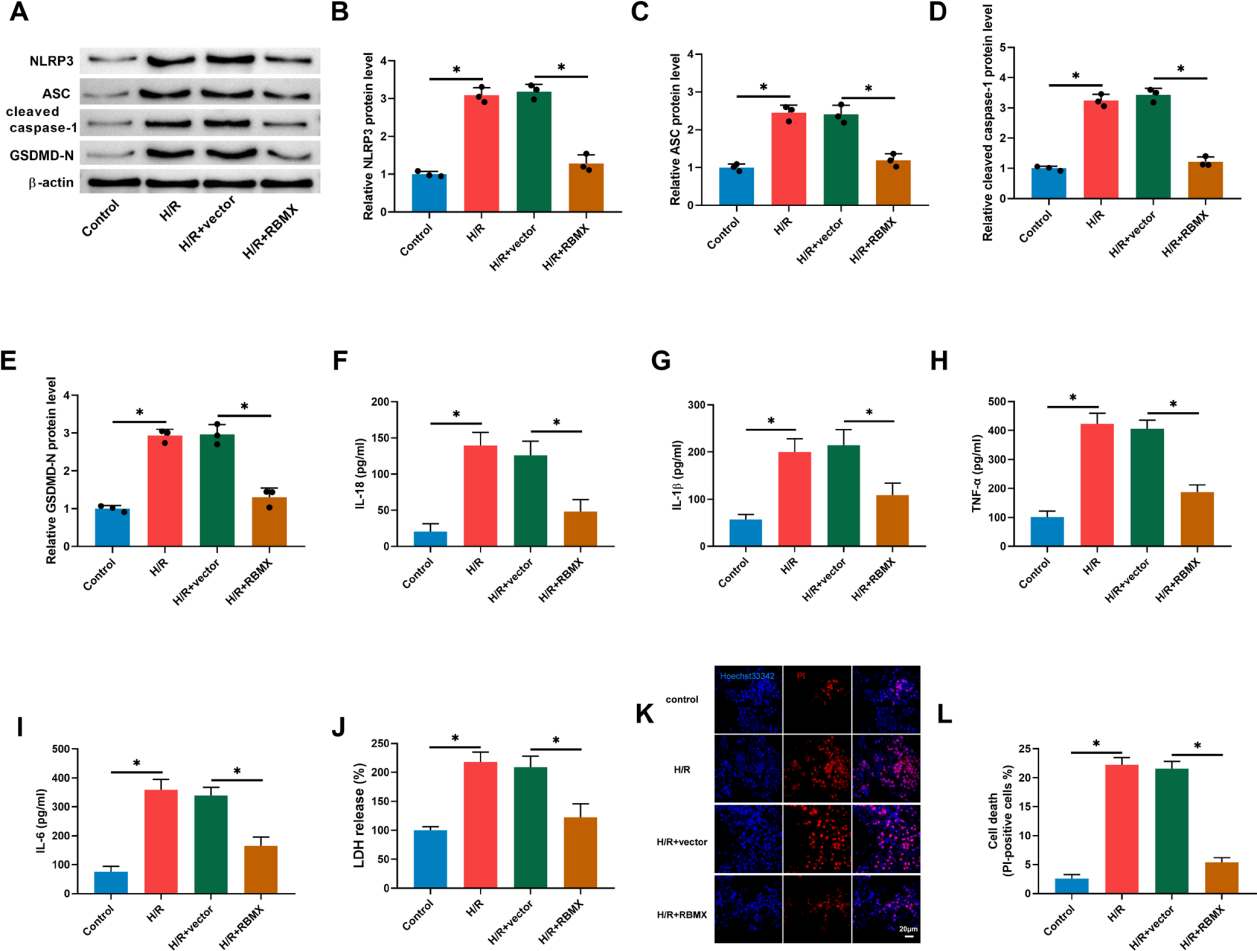
RBMX attenuates NLRP3 activation and pyroptosis in H/R‐induced HK‐2 cells. (A–E) Western blot analysis for expression levels of proteins involved in NLRP3 inflammasome activation, including NLRP3, ASC, cleaved caspase‐1, and GSDMD‐N. (F–I) ELISA for the secretion of inflammatory cytokines associated with pyroptosis, including IL‐18, IL‐1β, TNF‐α, and IL‐6. (J) LDH release. (K, L) PI staining. * *p* < 0.05.

Previous studies have indicated that NLRP3 inflammasome activation can result in GSDMD‐mediated pyroptosis [17, 18]. Therefore, we examined the impact of RBMX on NLRP3 inflammasome activation and pyroptosis in HK‐2 cells under H/R conditions. Our findings revealed that H/R treatment led to a notable rise in the expression levels of NLRP3, ASC, cleaved caspase‐1, and GSDMD‐N, which were reversed by RBMX overexpression (Figure 2A–E). Our data showed that the elevated levels of IL‐18, IL‐1β, TNF‐α, and IL‐6 in H/R‐treated HK‐2 cells were mitigated by RBMX overexpression (Figure 2F–I). LDH release and cell death were increased in cells after H/R treatment, which were reversed by RBMX overexpression (Figure 2J–L). These results suggest that RBMX overexpression mitigates H/R‐induced NLRP3 inflammasome activation and GSDMD‐mediated pyroptosis in HK‐2 cells.

### 3.3 RBMX Regulation of NLRP3 Acetylation Through m6A Modification of SIRT3

**FIGURE 3:**
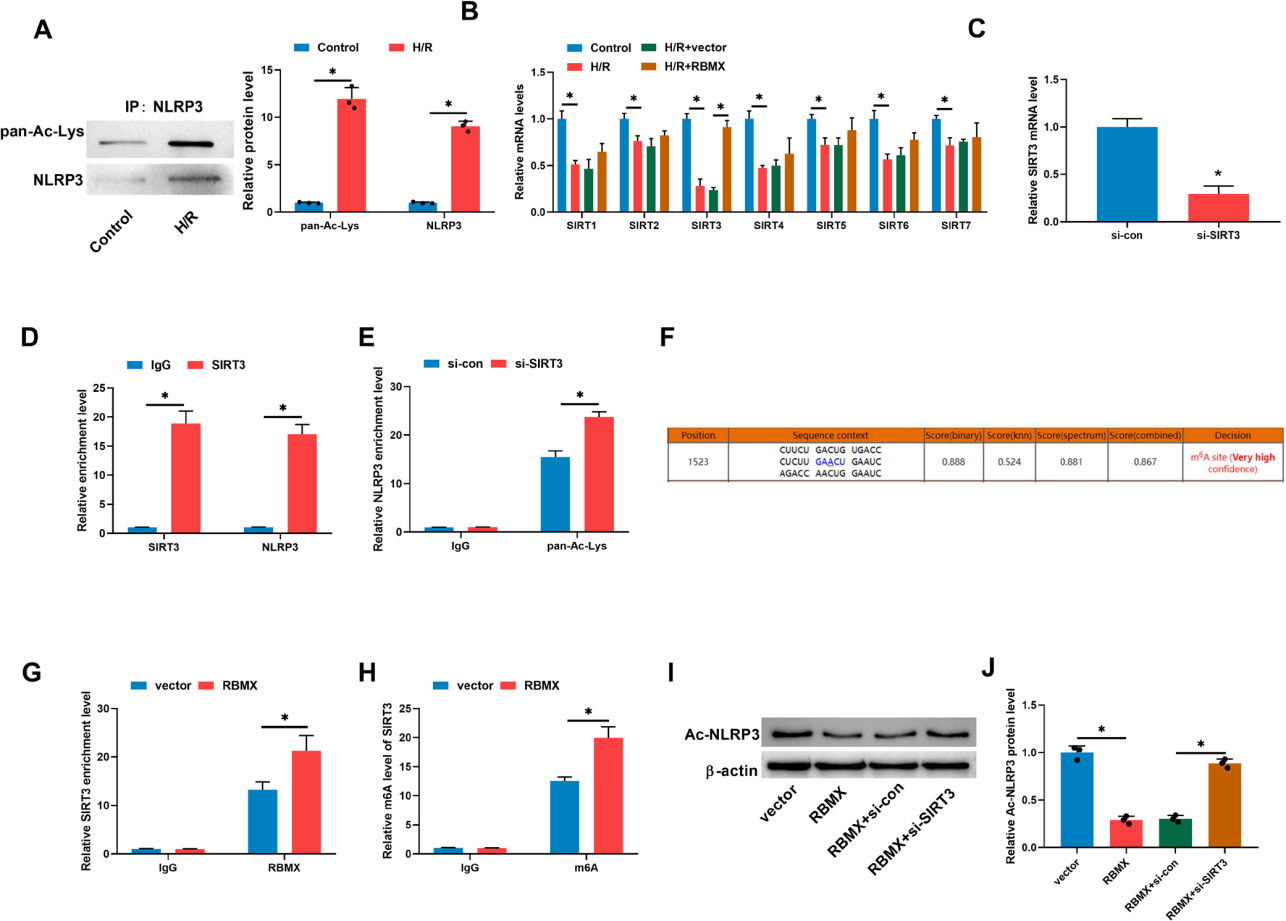
RBMX regulated NLRP3 acetylation through SIRT3. (A) IP assay indicated that acetylation of NLRP3 was significantly elevated in HK‐2 cells with H/R treatment. (B) The mRNA levels of SIRTs (SIRT1‐7) in HK‐2 cells. (C) Cells were transfected with si‐SIRT3 for SIRT3 silencing. (D) IP assay with anti‐SIRT3 for detecting the enrichment levels of SIRT3 and NLRP3. (E) IP assay with anti‐pan‐Ac‐Lys for detecting the enrichment level of NLRP3. (F) Potential of m6A sites of SIRT3 by SRAMP database. (G) RIP assay with anti‐RBMX for detecting the enrichment level of SIRT3. (H) MeRIP assay with anti‐m6A for detecting the mRNA level of SIRT3. (I, J) Western blot analysis for detecting the effects of RBMX and SIRT3 on acetylated NLRP3 expression. * *p* < 0.05.

Because acetylation of NLRP3 is crucial for full activation of the NLRP3 inflammasome, we investigated the effects of RBMX on NLRP3 acetylation. As shown in Figure 3A,H/R significantly increased NLRP3 acetylation in HK‐2 cells. It is evident that SIRTs are necessary for NLRP3 acetylation; hence, we analyzed the mRNA levels of various SIRTs (SIRT1‐7) in HK‐2 cells to determine the key SIRT protein. As shown in Figure 3B, SIRT3 mRNA levels markedly decreased in H/R‐treated HK‐2 cells and increased in RBMX‐overexpressing cells. Subsequently, cells were transfected with si‐SIRT3 for SIRT3 silencing to further explore the role of SIRT3 (Figure 3C). An IP assay using an SIRT3 antibody effectively precipitated SIRT3 and NLRP3 (Figure 3D). Additionally, SIRT3 knockdown elevated NLRP3 acetylation (Figure 3E). These findings suggested that SIRT3 might be associated with the acetylation of NLRP3.

To determine the m6A modification of SIRT3, the potential m6A sites of SIRT3 were predicted using the SRAMP database (Figure 3F). RBMX overexpression also increased SIRT3 expression (Figure 3G). The MeRIP assay showed that RBMX overexpression directly increased the m6A levels of SIRT3 mRNA (Figure 3H). Moreover, RBMX overexpression decreased NLRP3 acetylation in HK‐2 cells, which was reversed by si‐SIRT3 (Figure 3I,J). These findings suggest that RBMX might be involved in the m6A modification of SIRT3 and thus suppresses the acetylation of NLRP3.

### 3.4 Effects of SIRT3 Knockdown on RBMX‐Mediated Cell Proliferation

**FIGURE 4:**
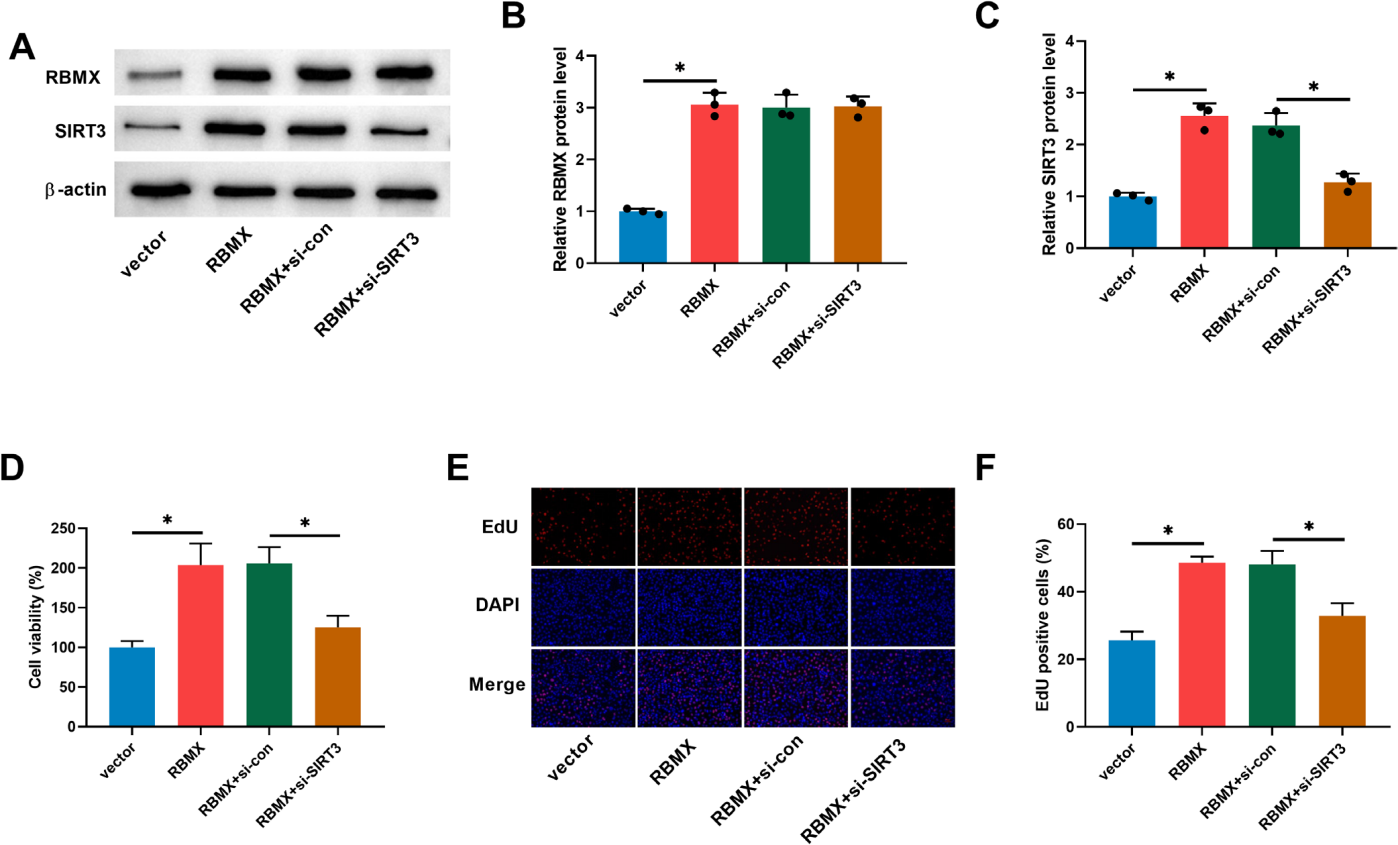
SIRT3 knockdown reversed the effect of RBMX on cell proliferation. (A–C) Western blot analysis for detecting the effect of RBMX on SIRT3 expression and assessing the transfection efficiency of si‐SIRT3. (D) CCK‐8 assay for detecting the effect of si‐SIRT3 on cell viability of RBMX‐overexpressing cells. (E, F) EdU for examining the effect of si‐SIRT3 on cell proliferation of RBMX‐overexpressing cells. * *p* < 0.05.

We investigated the involvement of SIRT3 in mediating the effects of RBMX. Western blotting indicated that SIRT3 expression was significantly upregulated in RBMX‐overexpressing cells but downregulated after transfection with si‐SIRT3 (Figure 4A–C). Furthermore, CCK‐8 and EdU assays showed that RBMX‐induced increases in cell viability and proliferation of H/R‐treated HK‐2 cells were reversed by si‐SIRT3 (Figure 4D–F).

### 3.5 Effects of SIRT3 Knockdown on RBMX‐Mediated NLRP3 Inflammasome Activation and Pyroptosis in H/R‐Treated HK‐2 Cells

**FIGURE 5:**
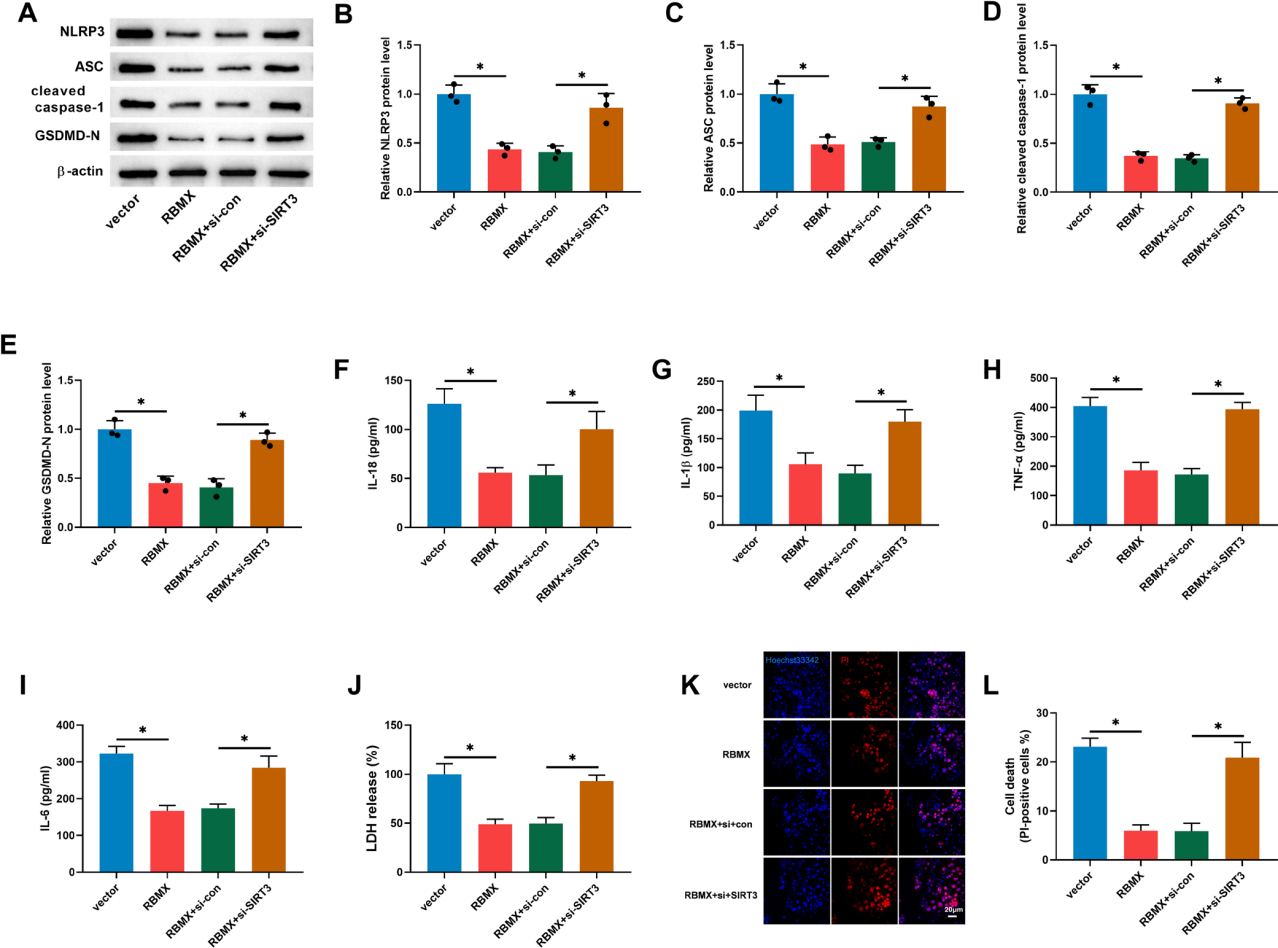
SIRT3 knockdown reversed the effects of RBMX on NLRP3 activation and pyroptosis. (A–E) RBMX‐caused decrease in expression levels of NLRP3, ASC, cleaved caspase‐1, and GSDMD‐N was reversed by si‐SIRT3. (F–I) RBMX‐caused decrease in IL‐18, IL‐1β, TNF‐α, and IL‐6 levels was reversed by si‐SIRT3. (J) LDH release. (K–L) PI staining. * *p* < 0.05.

The role of SIRT3 in mediating the effects of RBMX on NLRP3 inflammasome activation and pyroptosis was investigated. Western blotting results revealed that RBMX reduced the expression levels of NLRP3, ASC, cleaved caspase‐1, and GSDMD‐N in H/R‐treated HK‐2 cells, a trend reversed by si‐SIRT3 (Figure 5A–E). ELISA results indicated that the reduced levels of IL‐18, IL‐1β, TNF‐α, and IL‐6 in RBMX‐overexpressing cells were elevated following transfection with si‐SIRT3 (Figure 5F–I). Reduced LDH release and cell death by RBMX overexpression were attenuated due to SIRT3 silencing (Figure 5J–L). These findings suggest that SIRT3 is involved in mediating the inhibitory effects of RBMX on NLRP3 inflammasome activation and pyroptosis.

### 3.6 Improvement of Renal Injury in Mice by RBMX

**FIGURE 6:**
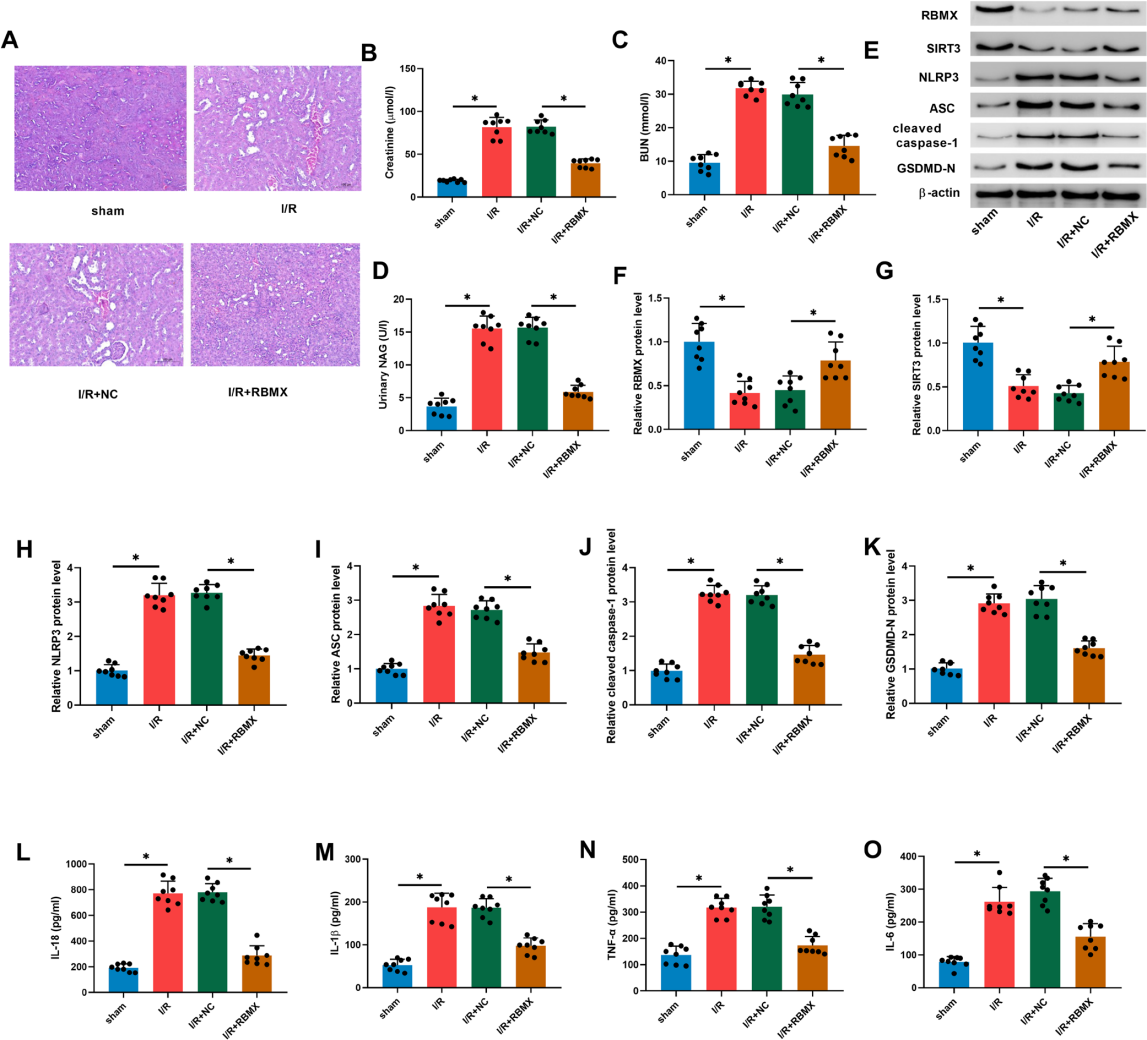
RBMX attenuated renal IRI and suppressed NLRP3 activation and pyroptosis in vivo. A recombinant Adeno‐Associated Virus (AAV) encoding the RBMX gene was administered through renal vein injection. (A) Representive HE staining images in kidney tissues. (B–D) Serum creatinine, BUN, and urinary NAG levels were detected using ELISA. (E–K) RBMX overexpression elevated the expression levels of RBMX and SIRT3 and suppressed the expression levels of NLRP3, ASC, cleaved caspase‐1, and GSDMD‐N. (L–O) RBMX overexpression reduced the serum levels of TNF‐α, IL‐1β, IL‐6, and IL‐18 in I/R mice. * *p* < 0.05.

The protective effects of RBMX against renal injury were examined in vivo. Histopathological analysis of the kidney tissues revealed that RBMX overexpression ameliorated tubular dilatation, reduced intratubular cast formation, and alleviated vacuole‐like degeneration of epithelial cells (Figure 6A). In the I/R group, creatinine, BUN, and NAG levels significantly increased, whereas RBMX overexpression decreased their levels in I/R mice (Figure 6B–D). Moreover, Western blot analysis showed that RBMX and SIRT3 expression levels were downregulated in I/R mice and upregulated in the RBMX overexpression group (Figure 6E–G). The expression levels of NLRP3, ASC, cleaved caspase‐1, and GSDMD‐N increased in I/R mice; however, these proteins were inhibited by RBMX overexpression (Figure 6H–K). Additionally, RBMX overexpression attenuated the elevated serum levels of IL‐18, IL‐1β, TNF‐α, and IL‐6 in I/R mice (Figure 6L–O). These findings suggest that RBMX confers a protective effect against renal I/R injury in vivo.

### 3.7 EZH2‐Associated H3K27me3 Enrichment at the RBMX Promoter

**FIGURE 7:**
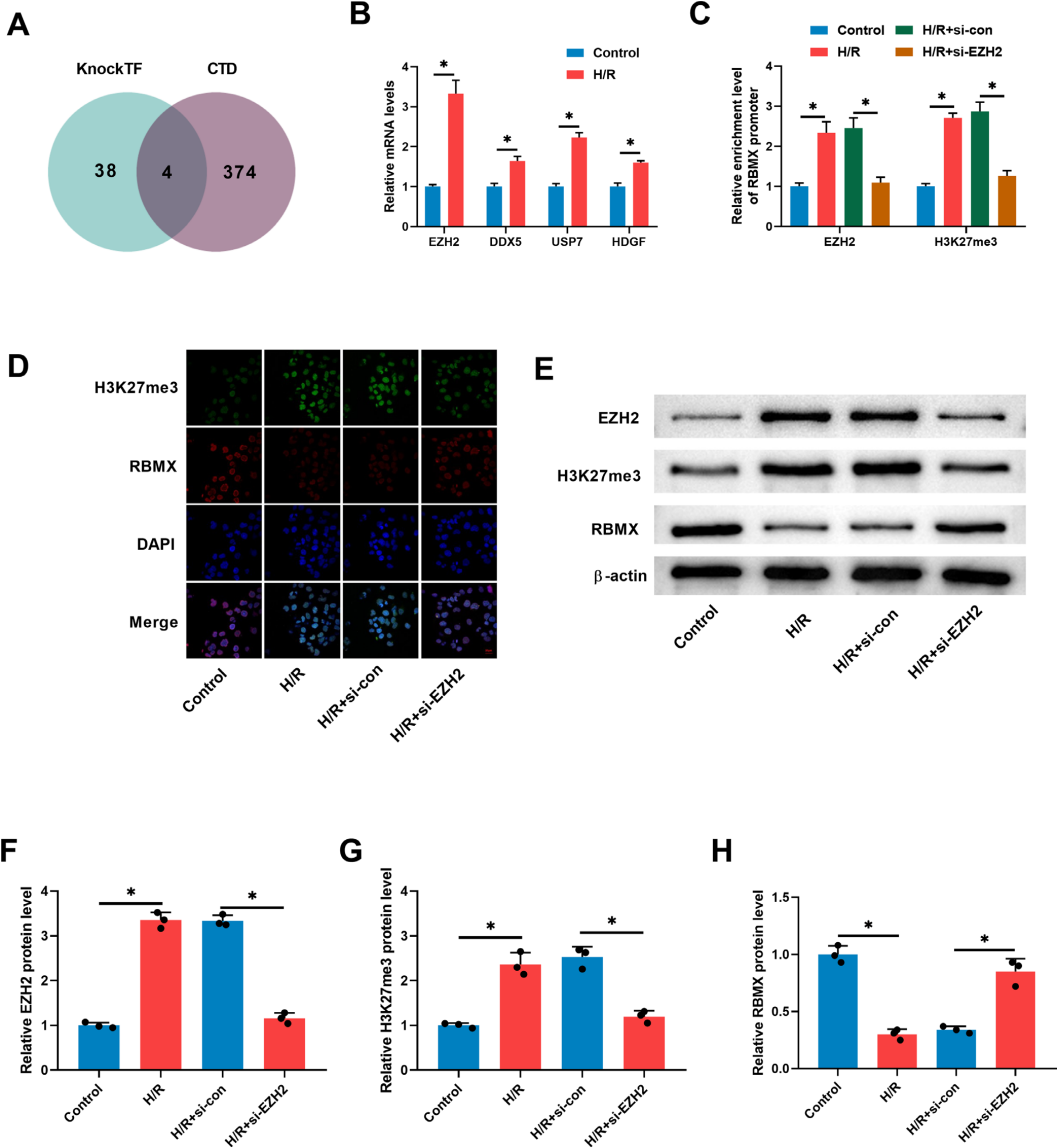
EZH2 mediated the H3K27me3 modification of RBMX and thus regulated RBMX expression. (A) Venn analysis for selecting the potential upstream genes of RBMX from knockTF and CTD database. (B) RT‐PCR for detecting the mRNA levels of 4 overlapping genes, EZH2, DDX5, USP7, HDGF in H/R‐treated HK‐2 cells. (C) ChIP assay for detecting the enrichment levels of EZH2 and H3K27me3 in the promoter region of RBMX. (D) IF staining for detecting the levels of RBMX and H3K27me3. (E–H) Western blot analysis for detecting the expression levels of EZH2, RBMX, and H3K27me3 to confirm the EZH2‐mediated H3K27me3 modification of RBMX. * *p* < 0.05.

To identify the upstream mechanism governing RBMX regulation, Venn analysis was performed using data from the knockTF and CTD databases. As shown in Figure 7A, four overlapping genes–EZH2, DDX5, USP7, and HDGF–were identified. Subsequently, we observed that EZH2 was significantly upregulated in H/R‐treated HK‐2 cells compared with the other three genes (Figure 7B). In addition, the ChIP assay showed that the enrichment levels of EZH2 and H3K27me3 in the promoter region of RBMX increased in H/R‐treated HK‐2 cells. However, EZH2 knockdown significantly reduced the enrichment of EZH2 and H3K27me3 (Figure 7C). IF staining showed that RBMX levels were decreased and H3K27me3 levels were increased in the H/R group. The alterations in RBMX and H3K27me3 levels were restored upon EZH2 knockdown (Figure 7D). Moreover, Western blot analysis showed that the expression levels of EZH2 and H3K27me3 were significantly increased, and RBMX expression was decreased in H/R‐treated HK‐2 cells. EZH2 knockdown reversed the changes in the expression levels of EZH2, H3K27me3, and RBMX (Figure 7E–H). These results imply EZH2‐associated H3K27me3 enrichment at the RBMX promoter and EZH2 may contribute to RBMX downregulation.

## 4 Discussion

**FIGURE 8:**
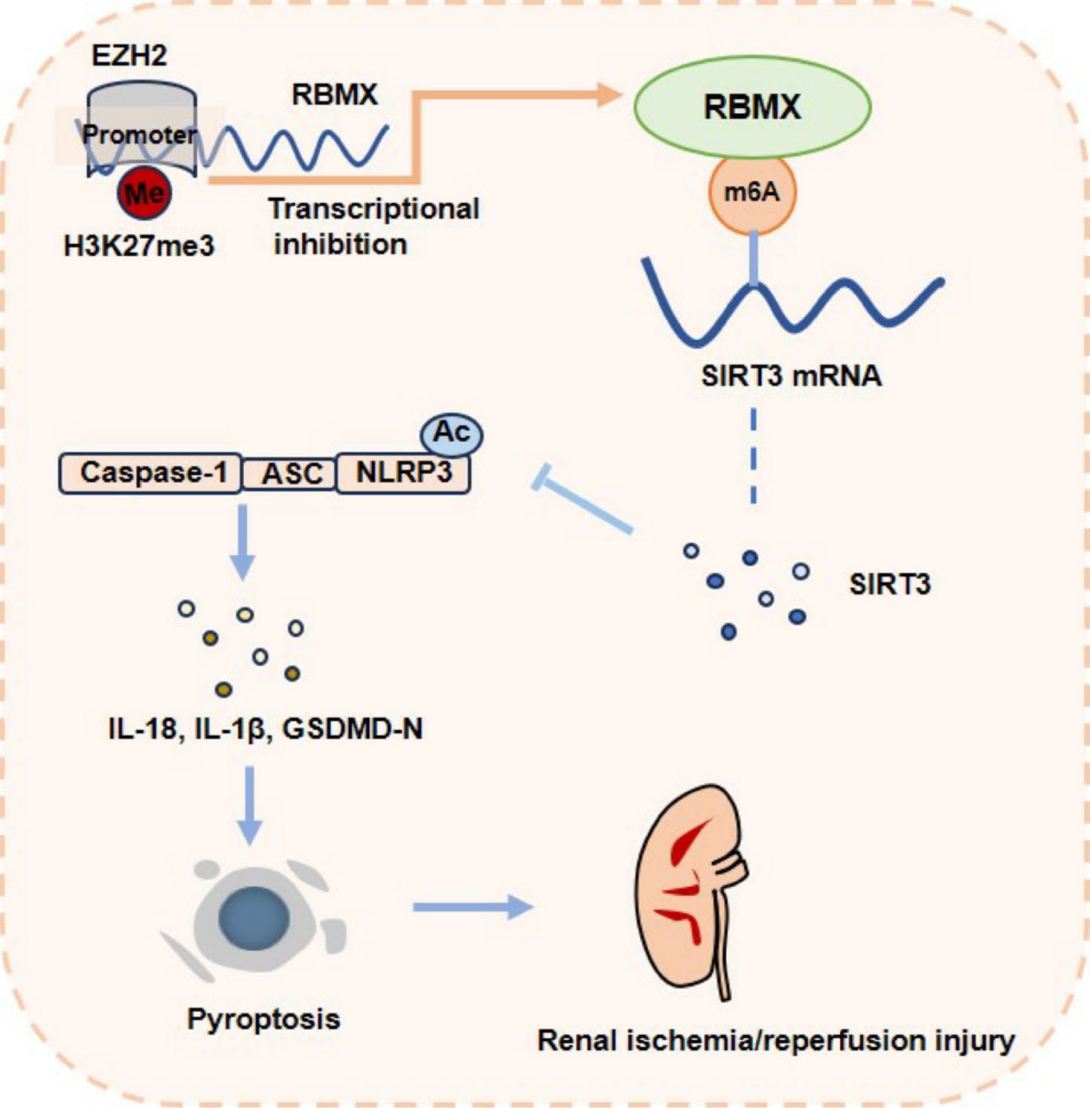
Mechanisms underlying the roles of RBMX in IRI. EZH2 mediated the H3K27me3 modification of RBMX and thus negatively regulated RBMX expression. RBMX regulated the m6A methylation of SIRT3, which inhibited NLRP3 inflammasome activation through regulating NLRP3 acetylation and eventually preventing pyroptosis.

A previous study showed that RBMX is downregulated in patients with ischaemic stroke [12]; however, the underlying molecular mechanism has not been reported. Consistent with a previous study, we found that RBMX was significantly downregulated in ischaemic mice and in vitro H/R‐treated HK‐2 cells. We further provided evidence suggesting that RBMX attenuated renal IRI possibly by regulating SIRT3/NLRP3 inflammasome activation‐mediated pyroptosis. The NLRP3 inflammasome is a cytosolic multiprotein heteromeric complex comprising the intracellular receptors NLRP3, ASC, and pro‐caspase‐1 [19]. Upon NLRP3 inflammasome activation, caspase‐1 cleaves and activates pro‐IL‐1β, pro‐IL‐18, and gasdermin D (GSDMD), leading to the production of mature IL‐1β and IL‐18, along with the N‐terminal cleavage product of GSDMD (GSDMD‐N) [18]. These processes initiate a novel inflammatory cell death mechanism known as pyroptosis [18]. Inhibiting NLRP3 inflammasome activation can prevent pyroptosis and may represent an effective approach for managing inflammatory conditions [20, 21]. In the present study, we found that RBMX overexpression markedly reduced NLRP3 inflammasome activation and pyroptosis in H/R‐exposed HK‐2 cells and a renal IRI mouse model (Figure 8). These findings suggest that RBMX may shield against renal IRI by suppressing NLRP3 inflammasome activation and pyroptosis.

NLRP3 undergoes acetylation and deacetylation by SIRTs [22]. Moreover, the acetylation of NLRP3 is pivotal for the assembly and activation of the NLRP3 inflammasome [22]. Consistent with prior studies, we observed that SIRT3 is implicated in the regulation of NLRP3 acetylation in H/R‐exposed HK‐2 cells. Previous studies have indicated that SIRT3 is an m6A‐modified protein involved in the progression of renal IRI [23, 24]. We discovered that the m6A regulator RBMX controlled m6A methylation of SIRT3. Knockdown of SIRT3 reversed the effects of RBMX on proliferation, NLRP3 inflammasome activation, and pyroptosis of H/R‐treated HK‐2 cells. These findings suggest that SIRT3 mediates the regulatory effects of RBMX on the subsequent acetylation and activation of NLRP3 and pyroptosis.

EZH2 is an enzymatic catalytic subunit of Polycomb repressive complex 2 (PRC2), which modifies downstream target gene suppression by trimethylating Lys‐27 in histone 3 (H3K27me3) [25]. Previous studies have shown that EZH2 is involved in IRI, including myocardial and cerebral IRI [26, 27, 28]. Notably, EZH2 was found to play an important role in AKI and CKD [29]. In AKI mice and patients, EZH2 and H3K27me3 were significantly upregulated in renal tissue [29]. Deletion of EZH2 significantly attenuates pathological lesions in I/R injury and enhances renal function in mice [29]. Furthermore, inhibition of EZH2 substantially suppresses oxidative stress and pyroptosis in H/R‐induced HK‐2 cells in vitro and in a murine renal IRI model [30]. Hence, targeted EZH2 inhibition could represent a promising therapeutic approach to alleviate renal IRI in AKI. This study explores the involvement of EZH2 in renal IRI, revealing that EZH2‐associated H3K27me3 may be enriched at the RBMX promoter and is correlated with RBMX expression in H/R‐treated HK‐2 cells. This implies that EZH2 may be involved in an upstream regulator of RBMX in renal IRI.

However, there are some limitations in the current study. Firstly, the current experiments were mainly performed in HK‐2 cells but not in primary cells. Many molecular responses in HK‐2 cells do not fully reflect human AKI biology. Hence, the function of RBMX should be explored in primary cells in the further study. Moreover, the regulatory network was only confirmed in vitro, and the claimed pathway remains mostly speculative. The in vivo mechanistic validation should be performed in future. Furthermore, the absence of bidirectional rescue experiments or pharmacologic modulators weakened the causal claims. To better understand the regulatory network, more work would be performed in future.

In conclusion, RBMX might regulate NLRP3 inflammasome activation and pyroptosis possibly by modulating m6A methylation of SIRT3, which might be associated with EZH2‐associated H3K27me3 modification. Targeting the potential EZH2/RBMX/SIRT3 axis might represent a novel therapeutic approach to prevent NLRP3 inflammasome activation and pyroptosis in renal IRI.

## Conflicts of Interest

The authors declare no conflicts of interest.

## Data Availability

The data that support the findings of this study are available from the corresponding author upon reasonable request.
